# Identifying mechanisms by which social determinants of health impact TB diagnostic evaluation uptake in Uganda: a qualitative study

**DOI:** 10.1186/s12939-025-02437-y

**Published:** 2025-03-14

**Authors:** Talemwa Nalugwa, Kristi Sidney Annerstedt, Sarah Nabwire, Nora S. West, Jillian L. Kadota, Salla Atkins, Adithya Cattamanchi, Knut Lönnroth, Achilles Katamba, Priya B. Shete

**Affiliations:** 1 World Alliance for Lung and Intensive Care Medicine in Uganda (WALIMU), Kampala, Uganda; 2 Department of Global Public Health, Karolinska Institutet, WHO TB Collaborating center, Stockholm, Sweden; 3 Division of Pulmonary and Critical Care Medicine, University of California San Francisco, San Francisco, CA USA; 4 Global Health and Development, Faculty of Social Sciences, Tampere University, Tampere, Finland; 5 Clinical Epidemiology & Biostatistics Unit, Department of Medicine, Makerere University College of Health Sciences, Kampala, Uganda

**Keywords:** Qualitative research, Tuberculosis, Social determinants of health, COM-B model

## Abstract

**Background:**

Social and structural determinants of health (SDoH) are associated with tuberculosis (TB) outcomes but often unaddressed in TB care programs. We sought to describe the mechanism by which SDoH impact completion of TB diagnostic evaluation in Uganda using an implementation science framework rooted in behavioral theory.

**Methods:**

Trained research staff interviewed 24 purposively sampled adults undergoing TB diagnostic evaluation at six community health centers in Uganda between February-August 2019. Framework analysis was used to construct themes linked to SDoH across the TB diagnostic evaluation cascade of care. Themes were then mapped to domains of the capability, opportunity, and motivation behavior change model (COM-B).

**Results:**

Barriers related to SDoH were identified across the diagnostic evaluation cascade of care and associated with domains central to uptake of testing and treatment. These included: limited knowledge about TB diagnosis and treatment (psychological capability); low socioeconomic status and competing financial priorities (physical opportunity); internalized and anticipated stigma of TB diagnosis, lack of social support programs and limited social support/social capital (social opportunity, reflective motivation); trust (or distrust) in the government health facility to provide quality care (reflective motivation); and fear and shame about worsening poverty (automatic motivation). Facilitators to engagement with the TB cascade of care included encouragement from friends and family to seek testing (automatic motivation, social opportunity), and trust that healthcare providers were acting in their best interests (social opportunity).

**Conclusions:**

Biomedical interventions alone are unlikely to address the spectrum of SDoH-related barriers to equitable completion of TB diagnostic evaluation. Linking barriers to a behavior change model such as COM-B may help guide the design and evaluation of appropriate people-centered strategies that integrate social and economic supports with clinical and public health programs or services.

**Supplementary information:**

The online version contains supplementary material available at 10.1186/s12939-025-02437-y.

**Table:** 

Text box 1. Contributions to the Literature (98/100 words)
• Existing evidence on social determinants of health (SDoH) related to tuberculosis (TB) describes how determinants increase risk of disease. Evidence on the influence of TB-related SDoH on capacity to engage in activities for seeking care and completing diagnostic evaluation is critically needed
• This study illustrates the application of a behavior change framework to qualitative data to map the mechanism by which SDoH may affect access, uptake, and completion of TB diagnostic evaluation.
• Evaluating the behavioral effect of social and structural determinants of health can reveal potential targets for interventions to address the root causes of disparities in TB

## Background

Tuberculosis (TB) continues to be a global health crisis, with an estimated 10.6 million new cases and 1.6 million deaths in 2021 [1]. ​​While TB treatment is readily available, social, structural, and behavioral factors impact care seeking and completion of TB treatment [2, 3]. Social determinants of health (SDoH)– such as food insecurity, poor housing and environmental conditions, geographic and cultural barriers to health care access, and limited health literacy– are common in low and middle income countries where TB-burden is high and contribute to a lower likelihood of TB diagnosis and treatment initiation [4–7]. Studies have further demonstrated that SDoH play a role in both risk of acquiring TB and poor treatment outcomes and mortality [2, 8–12]. However, the mechanisms by which these social and structural determinants influence the behaviors necessary for individuals to complete TB related care activities remains largely unknown. Much of the research conducted at the intersection of TB and SDoH to date has established that these determinants decrease an individual’s capacity to seek care and to complete TB diagnostic evaluation, particularly in low-income, high TB-burden settings [2, 13]. However, most of this research has been descriptive in nature. Evidence from HIV research demonstrates that SDoH play a key role in outcomes that depend upon affected individuals engaging in specific activities or behaviors including HIV prevention, acquisition, testing, and adherence to treatment [14–17]. Largely lacking and yet critically needed is clear evidence on the behavioral influence of TB-related SDoH and their role in engagement in TB diagnostics and care.

Uganda is a low-income setting with a high burden of TB [18] and has an estimated TB incidence rate of 199 per 100,000 population [1]. Pre-treatment loss to follow-up among people diagnosed with TB in Uganda is approximately 20% [19–21]. Treatment success rates in Uganda vary by region but are generally estimated to be less than 80% [1, 22, 23]. While Uganda, like all high-burden counties, has robust access to free TB diagnostics and treatment, the persistent high burden of TB demonstrates that diagnostics and treatment alone do not sufficiently address the needs of people with TB.

This study aimed to (1) identify and describe the social and structural barriers and facilitators of completing TB diagnostic evaluation in a high-TB burden, low-resourced setting, and (2) map these onto the behavioral COM-B model, an implementation science framework to inform the development of people-centered TB care interventions. Better understanding of the mechanisms by which SDoH impacts behaviors related to TB care is first needed to develop targeted interventions and implementation strategies that address the most influential social and economic determinants of poor TB outcomes.

## Methods

### Study design and setting

An exploratory qualitative approach with individual in-depth interviews was chosen to understand potential social barriers and/or facilitators for completing TB diagnostic evaluation among people presenting with a cough at community health centers. An additional file shows the Consolidated criteria for Reporting Qualitative (CORE-Q) [24] research as applied to this study [see Additional file 1].

The study took place in eight peri-urban and rural public run health centers (level IV) from six different districts in Uganda as part of an ongoing study [25]. These health centers are governed by the Ministry of Health (MOH) at the national level. Clinical services are provided at these health centers in accordance with Ministry of Health (MoH) regulations, by a team led by a medical doctor supported by clinical officers (a trained primary care health worker who is not a physician), qualified nurses and other health workers. The services provided at level IV centers include diagnostics/laboratory services, maternal health care, specialized clinics including HIV and TB care, and limited inpatient facilities. Individuals at risk for TB access screening and diagnostics services by receiving a laboratory-based sputum test. Seven of the eight included health centers offer on-site molecular sputum-based TB diagnostic tests, GeneXpert MTB/Rif (Xpert, Cepheid, Sunnyvale USA) to eligible patients who have been referred to the laboratory, and all included health centers have the capacity to provide TB treatment to patients.

### Theoretical frameworks

The World Health Organization’s Conceptual SDoH Framework has been widely used to explore the impacts of social determinants in a wide variety of populations and across a spectrum of health conditions, including HIV, mental health, noncommunicable diseases, sexual and reproductive health, and COVID-19 [26–31]. The framework outlines how social, economic and political mechanisms, along with the health system, impact equity in health and well-being [32]. The framework demonstrates a complex interplay between how the socioeconomic and political context, structural and social determinants of health inequities (such as gender, social class, education and income) are bridged by intermediary determinants including material circumstances (living and working conditions, and availability of food), behavioral and biological factors (habits like smoking and other lifestyle habits), and psychosocial factors.

In addition to the SDoH Framework, we utilized the COM-B model for behavior change, an implementation science framework rooted in behavioral theory, which has been widely used for understanding the mechanisms that drive behavior [33]. COM-B posits that the interaction of ‘capability’ (*physical* - skills or *psychological* - knowledge), ‘opportunity’ (*physical* - environmental resources or *social* - societal influences) and ‘motivation’ (*automatic* - emotions or *reflective* - beliefs, intentions) result in a specific behavior [33]. Interventions aiming to change behavior need to influence one or more of these components. A strength of COM-B as a behavior change model is its validated linkage to both: (a) the Theoretical Domains Framework which describes intervention functions that would target behavioral barriers and facilitators; and (b) the Behavior Change Wheel (BCW), a synthesis of 19 frameworks of behavior change, that has policy categories and implementation strategies that support designing effective theory-based interventions [33].

When used together, we hypothesize that the SDoH and COM-B frameworks can elucidate the complex relationship between determinants and specific activities needed for operationalizing effective person-centered TB care strategies.

### Participant recruitment and sample

Participants were purposively selected by gender and age. Eligible participants at each of the six participating health centers were identified by laboratory staff or by research assistants when submitting sputum samples to the laboratory for TB diagnostic evaluation. Study staff liaised with health workers at the sites to identify eligible participants. Eligible participants were 18 years or older and had symptoms consistent with TB though not yet diagnosed nor currently on TB treatment. Eligibility was confirmed at the time of interview. All participants provided informed consent in Luganda or English. Sample size was guided by the principle of saturation [34]. Given the interviews were focused on a specific topic of interest and conducted among a homogenous group of participants (e.g. individuals accessing TB screening and diagnostics), we estimated 20–30 interviews would be appropriate to reach thematic saturation based on prior experience in conducting similar interviews.

### Data collection

A topic guide [see Additional file 2], informed by the WHO’s Social Determinants of Health Framework, was developed by the study team and used to guide interviews [32]. The theoretical structural and intermediary determinants of the framework were adapted into specific questions pertaining to the participants’ experience at the clinic, their socio-economic status and material and psychosocial circumstances, and behavioral and biological factors related to TB. The topic guide was piloted among two participants at one of the health centers and revised as needed. We conducted an additional three interviews after an initial 21 interviews in order to explore relevant topics raised from the previous interviews and identified during the data analysis.

Trained female research staff, T.N, and S.N., with bachelor’s degrees and extensive experience with qualitative interviewing conducted the interviews between December 2018 and August 2019. We invited 29 people and of these 24 consented to participate. The five who declined participation cited lack of time, were too unwell to participate in the interview or refused to participate. At the time of the interview, the interviewer introduced themselves to the participant and described the purpose of the study before administering the consent form which provided information about the study. Only the interviewer at participant were present for all interviews. All 24 interviews were conducted in the local language (Luganda) or English, audio-recorded and lasted up to one hour. A quiet space within the health center premises was agreed upon by both the interviewee and interviewer to maintain privacy. All interviews were translated (as necessary) and transcribed into English by an experienced team member and proofread for quality assurance by S.N. and T.N. Handwritten field notes about the interview process, impressions of the participant, success and challenges, and initial findings were recorded during interviews and reviewed during analysis.

### Reflexivity

The research team performing the study consisted of Ugandan and international social scientists, doctors, epidemiologists and implementation scientists with health system and TB expertise. The two research staff who participated in the data collection process were aware of the local contextual characteristics, had a strong command of the local language (Luganda), which was commonly used for interviewing, and were also fluent in English. Using open-ended qualitative inquiry, there were no specific expectations for findings, but it is possible that phrasing of questions and interviewers influenced participant responses. Transcription of the audio recordings was done by a professional transcriber with a social science background who was not part of the study team. The composition of the team included senior researchers with vast experience in qualitative research methodologies and other research staff who were early researchers utilizing qualitative methodologies. Data collectors engaged in reflexivity by describing their own positionality during research debriefings, including sharing personal experiences related to TB diagnostic evaluation and health care seeking and their prior professional experiences in health care delivery. Member checking to validate interpretation of data collected among the overall research team allowed study staff the opportunity to explore the ways in which positionality added nuance to study findings.

### Data analysis

Data-driven thematic framework analysis [35], a matrix based approach to structure and synthesize data, was led by the joint first authors (K.S.A. and T.N.) with input from the study team. K.S.A. developed a framework to index recurring concepts and ideas within the data. After discussion and review of the transcripts, the research team agreed on a set of codes forming the initial analytical framework. It reflected descriptions or ideas about perceptions of access to care, experiences of their health care visit, stigma related to the illness, and different support mechanisms for care. Each code was given a brief explanatory description of its meaning to provide consistency during the coding process. Two randomly selected interviews were coded with the analytical framework. After the initial codebook development and coding, codes were aligned to the SDoH framework constructs were developed and agreed upon by the analytic team. The final framework consisted of 35 codes, clustered into 6 concepts related to the TB diagnostic cascade. S.N. indexed all the interviews in Microsoft Word and K.S.A. and T.N. double indexed them to increase reliability. S.N. and K.S.A. charted the participants’ responses into the thematic matrix with Microsoft Excel. The data were viewed vertically and horizontally through the framework matrix by categories. Findings were compared across gender and age, with no meaningful differences found unless noted. Themes were constructed to highlight perceptions, behaviors, and experiences. J.K. and K.S.A. mapped the themes to the COM-B model components.

### Ethical considerations

The study was approved by the School of Medicine Research and Ethics Committee, Makerere University College of Health Sciences, the Uganda National Council for Science and Technology, and University of California San Francisco Committee on Human Subjects Research. The study information was verbally explained, and all participants provided written informed consent prior to the initiation of the interview. Participants discussed potentially sensitive topics and were reminded of their right to withdraw at any point of the study if they felt uncomfortable. None withdrew participation; however, two interviews were shortened when the participant appeared distracted or unwell.

## Results

**Fig. 1:**
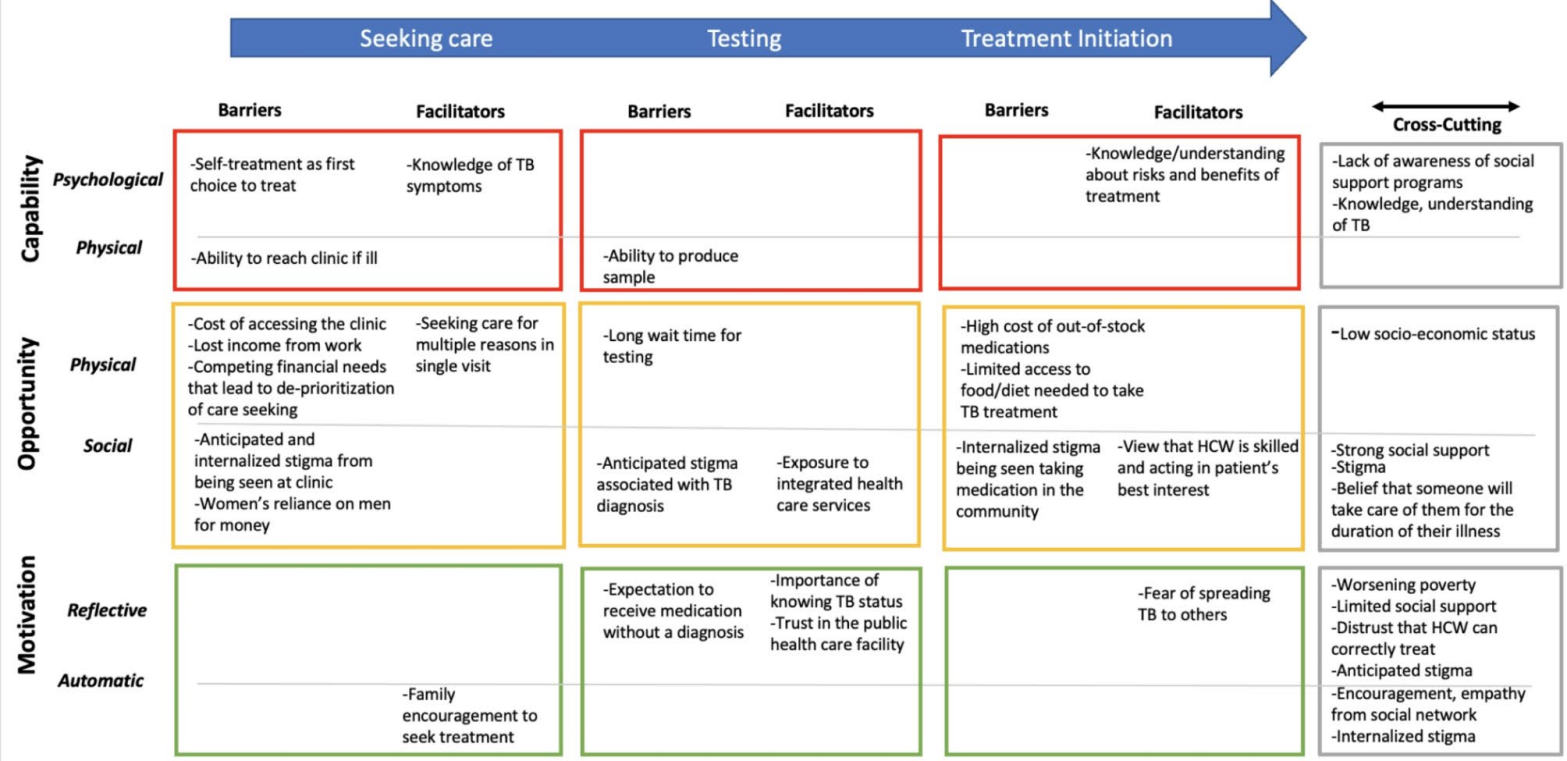
Barriers, facilitators, and cross-cutting factors to completing TB diagnostic evaluation along the cascade of care, mapped on to the COM-B Model Legend: This matrix represents the cross-referencing of themes elicited in interviews from a SDoH perspective and further mapped by which step of TB diagnostic evaluation the theme relates to (represented temporally by the blue arrow, X- axis), and how it influences behavior according to COM-B domains (Y-axis). Cross-cutting themes which affect all aspects of diagnostic evaluation are noted in the grey boxes at the far right

Participants included ten women and 14 men ranging from 19 years of age to over 60. Participants lived in a variety of rural (*n* = 13) and peri-urban (*n* = 11) areas. Demographics are presented in Additional File 3. Themes were present within the majority of SDoH domains and many related to socioeconomic status, income, and social support needs and policies. Psychosocial and health system factors that influenced TB diagnostic evaluation were also frequently noted by participants. When SDoH were mapped to the COM-B Framework (Fig. 1) the majority of domains represented impacted “Opportunity” (both social and physical) to engage in care seeking, testing, and treatment initiation. Opportunity in this framework refers to external factors in the physical and social environments that make the successful conduct of the behavior possible. Relevant themes and representative quotations within the SDoH constructs related to TB diagnostic evaluation are further presented with the COM-B constructs shown in the text with brackets–i.e., theme [*COM-B subconstruct*] along TB the diagnostic cascade of care.

### Cross-cutting barriers and facilitators along the TB diagnostic cascade of care

#### Socioeconomic position

*Low socio-economic status [physical opportunity]* and its negative impact on health was a pervasive cross-cutting theme throughout all interviews and particularly illustrated by this participant (age 49); *“If I had money*,* I think at the time I started coughing is when I would have come with my children…the thing in this world that makes us the most sick*,* even considering long term diseases*,* is lack of money…”.* Most participants also described limited economic resiliency and implied this impacted their decision and ultimately ability to seek care, get tested and start treatment.

Female participants described potential discrimination in public for cough, perceived as TB, which could impact their ability to obtain work. Further, female participants described potential social isolation that included a lack of childcare and interaction with their community. In comparison to the women, most male participants reported a different interpretation of a potential diagnosis of TB. While male participants did not as often describe feeling discriminated against for their TB status or symptoms (i.e., coughing in public), they discussed a sense of isolation that was associated with either their symptoms or the knowledge of having TB. However, similarly, much of this was explained as the result of the feeling that people in their social network would not want to interact with them based on TB status.

*Fear*,* shame and worry about worsening poverty [reflective motivation]* was described as compounded by low socio-economic status. Several participants reported feeling embarrassed and shameful when they could not afford medicines resulting in either not receiving care/treatment or pushing them further into poverty.

#### Social policies and education

*Lack of awareness of social support programs [psychological capability]*: Middle-aged participants (age: 35–53) were the most able to describe the available support mechanisms in their community. Otherwise, these programs were not discussed by other participants, including the youngest and oldest participants who had minimal awareness of these programs. Those who did discuss social support programs, particularly women, often described churches and community-based saving groups. When mentioned, participants described challenges when accessing community supported programs for social protection. For example, a 35-year-old male participant reported: *“You cannot go to a savings association if you have no savings there. The savings associations right now save and lend to each other*,* you understand? So you cannot go to the savings association to borrow when you are sick when I have not saved anything. They say you reap what you sow… If you have not saved anything with them*,* you cannot borrow from them.”*

#### Psychosocial factors

*Stigma [social opportunity*,* reflective motivation]*: Some participants reported both anticipated and internalized stigma. Men and women had different perspectives on how receiving a TB diagnosis would affect them. Men were more likely to discuss the potential impacts of stigma and thought most people in their immediate social support networks would judge and treat them negatively based on TB status, on an individual level. Female participants discussed less stigma but felt that it would impact them in their communities and were concerned about community-level stigma. This was described as reinforcing the need to ensure status was not disclosed, as expressed by one female participant: *“I do not bother them [people in the community] with the knowledge that maybe I am a TB patient. I do not want to be the laughing stock. You will tell them and they will go and broadcast this information in the village” Female*,* Age 28*.

*Strong social support networks [social opportunity*,* reflective motivation]*: Most participants described having support, encouragement and empathy from their family during their illness, a critical component to progressing through the care cascade, while also providing a strong social network as reported by one participant: *“I was reluctant to seek treatment but they [family members] encouraged me the most saying that it might not be the usual cough and that I should go to the hospital”.-Male*,* Age 42*.

Many participants also described a willingness to help members of their communities whether it was providing transport for a neighbor in labor or to collect medicines for someone who was ill. However, when they discussed their own social capital, they did not have an extensive network outside of their immediate family willing to offer help or that they would feel comfortable asking for help.

### Seeking care

#### Socioeconomic position

*Ability to reach the clinic and high cost of accessing the clinic [physical opportunity]*: Participants described issues in reaching the clinic driven by poor financial resources. Gaining access to transport (i.e., a bicycle, paid motorcyclists, or a car) was reported by some participants who lived in rural areas. Many participants from peri-urban areas described difficulties paying for transport to access testing. Some participants explicitly stated delaying seeking care due to lack of finances for transport and others said it would be difficult to find money to pay for multiple trips if their diagnostic evaluation or treatment required it. One young man clearly described his perception of the chain of events from not having money: *“If you don’t have money*,* you cannot do anything. Yes*,* you might get medicine for free but have no money for the transport. If you do not have transport money*,* which means you will also not get the treatment”. Male*,* age 35*.

#### Income

##### Lost income by not working [physical opportunity

Participants often reported the need to reserve an entire day for the clinic visit, resulting in loss of income for the day as many participants were not salaried workers and would not be able to perform any other income generating activities.

##### Competing financial needs and tasks in the household lead to de-prioritization of care-seeking [physical opportunity

Many participants described the financial needs of the household (e.g., caring for the children in terms of providing food, clothing, or school fees) superseded the care seeking for TB symptoms. One woman described how competing financial needs in the household and poor financial resources interplayed with one another *“I was supposed to come here [to the hospital] earlier but I had to dig to be able to get food for the children… I failed to make it because I did not have transport and the money I earned I used to buy the children food.”– Female*,* Age 26*.

#### Gender and socioeconomic position

Responses were gendered when it came to financial needs in the household as a barrier to care seeking: female participants described the need to maintain and support the household through domestic duties while, male participants reported fear of not being able to financially provide for the family. One male participant described this as follows: *“[Financial worries] can stop me from coming to hospital because I am the head [of the household] and if I do not work I will not eat or even my family will not survive so I decide to go and work to be able to secure something. That can stop you from going to hospital”– Male*,* 35*.

*Women’s reliance on men for money [social opportunity]*: Dependence on male counterparts was reported as having an impact on women’s ability to visit health centers, as women had to wait at home until the men provided funds for travel to the health center. On the other hand, some male participants said that women’s reliance on them to help pay for transport and other household related costs inhibited their own ability to seek care when needed, because they had to continue working to provide for the family and may contribute to why women often seek care faster and more frequently compared to men. As described by a 35-year-old male participant: *“When they (women) ask for money*,* however little*,* they have nowhere to get it and so she has to ask for the money from the man because they [women] do not have the ability to pay.”*

#### Health system factors

*Seeking care for multiple reasons in one visit [physical opportunity]*:. Some participants reported circumstances outside their own health/symptoms or current illness as the driving factor behind seeking care at the health facility. As described, reasons usually fell into one of three categories: (1) convenience (i.e., they had another activity in the area and took the opportunity to come to the facility), (2) a previously scheduled appointment for their HIV treatment, or (3) were caring for a sick child and decided to seek treatment as well. As reported by a male participant, *“…I brought a child that is supposed to get medicine today so I took the opportunity to get tested as well. I wanted to use the same opportunity you see sometimes we have to travel a long distance”. Male*,* 54*.

### Testing

#### Health system factors

##### Long wait time at the clinic to be tested [physical opportunity

While participants reported issues including long wait time and lack of communication regarding the diagnostic process, in general they were satisfied with their TB testing experience. Participants expressed that they expected to receive medication immediately upon seeing a healthcare provider without a formal diagnosis *[reflective motivation]*. While some understood the importance of receiving a diagnosis confirmed by the laboratory tests before being prescribed medication, they felt the amount of time spent waiting was excessive.

##### Exposure to integrated health care services [social opportunity

Participants with prior exposure to HIV care seemed to have greater TB knowledge and understanding of the importance of testing and treatment. A few participants who reported previous interactions with the health care facility through HIV care did not hesitate to seek care once TB symptoms emerged. They frequently cited the information provided by health care providers as the reason for trusting that it was important to seek care immediately.

*Trust in the public facility to test and treat appropriately [reflective motivation]*: Many participants reported seeking care, usually in the form of medicine, elsewhere before coming to the facility. Others described a preference and level of trust with the public health facility to test and treat their ailments appropriately. One participant described his rationale for coming to the public facility instead of a private one like this: *“Most of the time*,* if I have flu with no cough or a blood test is not required; I usually go to private facilities…they give you tablets and you don’t know what exact condition you are treating. However*,* if you come here and get tested*,* you know exactly what illness you are treating.”–* Male, Age 35.

### Treatment initiation

#### Health system factors

##### High cost of out-of-stock medications [physical opportunity

Many participants acknowledged that the activities related to receiving care (i.e., evaluation and diagnostics tests) at the health facility are free. However, participants reported medication stock-outs that would require them to pay out-of-pocket for another medication which they explicitly stated they could not afford. Thus, the excessive out-of-stock of medications, as described by participants, forced many patients to procure their medications at high personal cost.

*Perception Healthcare Worker (HCW) is skilled and acting in their best interest [social opportunity]*: Several participants reported their expectations for the visit were exceeded and there was a strong perception that the HCWs were testing and treating for the correct disease. As reported by a female participant: *“When I came [today] I thought they would just write the medicine for me without even checking my sputum but since they have examined the sputum I will know what I am suffering from…and that they will be treating the right thing.” Female*,* Age 26*.

#### Material circumstances

##### Limited access to food/diet to support continuation of medication [physical opportunity

Participants expressed concern regarding their ability to afford the enhanced diet recommended during treatment. This was discussed most frequently by participants who had previously been diagnosed with TB or had a relative who had been diagnosed with TB.

## Discussion

In this qualitative study, among people presenting for care with TB symptoms, we found most participants reported experiencing SDoH-driven challenges in the TB diagnostic evaluation process. Overall, participants reported a complex interconnection between multi-level social determinants of health and TB care seeking, testing, and treatment behavior. Care seeking delays were described as multifaceted and reflected the effect of social, financial, behavioral, psychosocial, and systemic determinants (Fig. 1). Our analysis notably found that the majority of the SDoH framework domains were relevant to TB diagnostic evaluation. When mapped to the COM-B framework, we found these SDoH domains influenced TB diagnostic and care seeking behavior by affecting the essential constructs of capability, opportunity, and motivation. While these results may seem overwhelming, by situating our findings within the COM-B model we can streamline them into improved intervention design and implementation as well as potential policy approaches to facilitate change. For example, while the breadth of SDoH domains noted in our analysis suggests no single determinant was responsible for influencing diagnostic evaluation, most all of these SDoH factors affected “opportunity” for accessing and completing TB care. Using the Behavior Change Wheel (BCW) [33], which translates sources of behavior (capability, opportunity, motivation) into potential implementation strategies, our results suggest that strategies which enable behavior, through environmental restructuring, education, incentivization and facilitated by enhanced environmental and social policies and planning, could mitigate barriers to TB diagnostic evaluation faced by individuals in this high TB burden, low-income setting [33].

In line with other studies that have assessed the barriers, burdens and challenges reported by individuals during TB diagnostic evaluation [36, 37], we found that cost tradeoffs are made in the beginning of the TB evaluation process as individuals decide to seek care and are significant barriers that mitigate the physical and social opportunities necessary for testing and treatment initiation as well as the reflective motivation required to adhere to care. During the diagnostic process, participants frequently reported loss of income, which in the Uganda setting is further complicated by the fact that more than half of all income generating activities are within the informal labor economy, and unemployment is also high [38]. Therefore, care seeking requires both actively deciding against at least one day of wages or identifying a source of money to seek care, and requires acceptance of added transport costs. Interventions such as social planning policies, including social protection or social development programs, could provide the opportunity necessary to offset costs that prevent individuals the opportunity to access TB diagnostics and could incentivize individuals to remain in care. Such programs often defray either direct costs associated with attending the clinic, such as cash transfers or transportation vouchers, or offset opportunity costs that often endanger basic needs, for example, money to purchase food and pay for school fees for children [39]. Cash transfer interventions have also shown significant promise as an approach that can mitigate barriers and motivate people to engage in the TB diagnostic evaluation cascade [40].

We found anticipated and internalized TB-related stigma to be significant factors that were well described and impacted participants in our study and undermined the social opportunity and reflective motivation central to seeking care, testing, and treatment initiation behaviors. TB-related stigma has been well documented as a significant factor in engagement in TB prevention, care seeking, and TB treatment [41–43]. It persists despite the known interplay with uptake of and adherence to TB care, particularly in high-burden settings. There is a dearth of well designed, evidence-based interventions to address TB-related stigma in this context. TB clubs or social support groups have been used to address internalized stigma in several settings, with modest impacts [44, 45]. However, these clubs are better suited to individuals who have already been initiated on treatment. Education, another tenant of the BCW intervention aligns with addressing social opportunity-related sources of behavior, and training to address motivation-influenced sources of behavior, may be a starting point to consider appropriate interventions to address TB-stigma in the TB diagnostic evaluation period. However any intervention focused on education will need to consider the extant literature and existing approaches that have had mixed success [45]. As anticipated and internalized stigma reinforce loss to follow up in TB care, interventions to address these factors remain of critical importance.

Our findings revealed that gender inequality contributed to the delays both men and women described as experiencing in their TB diagnostic evaluation journey by limiting their social opportunity to access care. For example, many women in our study said they deferred visiting the health center because they had to take care of children and provide food for them. Women with younger children reported they could not leave their children at home without any care. This traditional division of domestic labor gender roles caused many women to postpone health visits due to limited opportunity. Men described that missing a day of work because of their sickness could interrupt their ability to provide for the family and violate their roles as providers for the household. Because of the cultural structure of gender roles and limited access to finances, women depended on men to provide financial support to enable travel to the health center, eroding their opportunity to seek care and testing. These findings are in keeping with existing literature on the role of gender in TB diagnosis. A systematic review conducted in the African and Asian regions reported that women more than men were affected by financial, physical, stigma, and literacy related barriers in TB diagnostic evaluation [46]. While providing money and social support alone may not solve gender inequalities directly, it might allow health to be prioritized with all the other household needs for women, thereby reducing TB disparities.

Our study was the first in our setting to document SDoH as barriers and enablers to TB diagnostics evaluation when mapped onto a behavior change model. We were able to identify key drivers among the social determinants of health that could influence participant behavioral changes. However, this study was not without limitations. Despite efforts to purposively recruit this age group, representation from women in the age range of 60 years and over was lacking in our sample. We attempted to mitigate this limitation by interviewing an additional 3 participants with the goal of increasing the representation of this group. We recruited participants who were already present and engaging in TB diagnostic evaluation, which may mean we lack the perspectives of individuals who have yet to engage in this process. However, participants were able to speak to their decision-making process to seek care and the barriers and facilitators before they presented to the health center. While qualitative findings are not intended to be generalizable, our findings provide important insights that may be transferable to other high-burden TB settings. Additionally, our use of framework analysis and the familiarity of primary data analysts with the context in which the study was conducted lend to the trustworthiness of these findings. This methodology may be used to identify context specific social and structural barriers and facilitators to TB diagnostic evaluation or other aspects of TB prevention and care that can be targeted by interventions that address capability, opportunity, and motivation using additional implementation science tools.

## Conclusions

Our study demonstrates that even with widespread availability of TB diagnostic evaluation, biomedical interventions alone are not enough to address the significant number of SDoH-related barriers to completion of evaluation. SDoH substantially affect the capability, opportunity, and motivation of people to successfully engage in TB screening, testing, and treatment initiation. Linking these barriers to a behavior change model can help guide the design and evaluation of appropriate people-centered interventions, such as stigma reduction and promotion of social protection programs, that address social and structural determinants of health and can be particularly important for diseases of poverty like TB. Further evaluation of the relative weight of different SDoH and for whom, at each step in the diagnostic cascade, will yield additional insights into time points when interventions to address them are most critical. Validation of these approaches in different contexts, different key populations, and across the full cascade of TB care will support scalable social and economic interventions that mitigate the negative effect of many of these SDoH. Overall, our analysis of social and structural determinants and their influence on behavior may be useful in designing efficient person-centered interventions and policies that reduce early loss to TB care critical to treatment success.

## Electronic supplementary material

Below is the link to the electronic supplementary material.

Addtional File 1: Checklist

Additional File 2: Participant demographics

Additional File 3: Topic Guide

## Data Availability

De-identified textual data can be made available upon reasonable request to the corresponding author.

## Abbreviations

BCW: Behavior Change Wheel
COM-B: Capability, Opportunity, Motivation-Behavior
HCW: Healthcare Worker
MOH: Ministry of Health
NTLP: National Tuberculosis and Leprosy Programme
SDoH: Social Determinants of Health
TB: Tuberculosis
WHO: World Health Organization
