# Supplementary material for: Identifying mechanisms by which social determinants of health impact TB diagnostic evaluation uptake in Uganda: a qualitative study
Source: Int J Equity Health. 2025 Mar 14;24:73. doi: 10.1186/s12939-025-02437-y (PMC11909805; doi:10.1186/s12939-025-02437-y)
Supplement: Supplementary file 2 — Additional File 2: Participant demographics [file 12939_2025_2437_MOESM2_ESM.docx]

## **Additional File 2. Demographics**

Table 1: Characteristicsof the study participants n=24

| **Gender** | **Age Range** | **Health Center (HC)** | **Peri-Urban/Rural** |
| --- | --- | --- | --- |
| F | 19-29 | HC-1 | Peri-urban |
| F | 19-29 | HC-3 | Peri-urban |
| F | 19-29 | HC-5 | Rural |
| F | 19-29 | HC-5 | Rural |
| F | 19-29 | HC-5 | Rural |
| F | 30-39 | HC-3 | Peri-urban |
| F | 40-49 | HC-4 | Rural |
| F | 50-59 | HC-6 | Rural |
| F | 50-59 | HC-6 | Rural |
| F | 60+ | HC-3 | Peri-urban |
| M | 19-29 | HC-1 | Peri-Urban |
| M | 19-29 | HC-3 | Peri-urban |
| M | 19-29 | HC-3 | Peri-urban |
| M | 19-29 | HC-5 | Rural |
| M | 30-39 | HC-1 | Peri-urban |
| M | 30-39 | HC-1 | Peri-urban |
| M | 30-39 | HC-6 | Rural |
| M | 40-49 | HC-2 | Rural |
| M | 40-49 | HC-6 | Rural |
| M | 50-59 | HC-3 | Peri-urban |
| M | 50-59 | HC-4 | Rural |
| M | 60+ | HC-3 | Peri-urban |
| M | 60+ | HC-5 | Rural |
| M | 60+ | HC-6 | Rural |
