# Supplementary material for: Identifying mechanisms by which social determinants of health impact TB diagnostic evaluation uptake in Uganda: a qualitative study
Source: Int J Equity Health. 2025 Mar 14;24:73. doi: 10.1186/s12939-025-02437-y (PMC11909805; doi:10.1186/s12939-025-02437-y)
Supplement: Supplementary file 3 — Additional File 3: Topic Guide [file 12939_2025_2437_MOESM3_ESM.docx]

**Additional File 3. Social Barriers Topic Guide**

**General Questions about Participant and the Clinic**

1. Tell me about why you came to the clinic today. Probe: What symptoms are you having? What are you worried about?

2. Tell me about your visit today? How was it?

Probes: Was this a typical clinic visit for you? Y/N

How Did your experience meet your expectations about what would happen? Y/N

- What activities did you engage in?
- What kinds of staff or providers did you see?

3. How certain were you that you would come to clinic today? Probe: What things in your life made you consider not coming? What things in your life pushed you to come?

**Socioeconomic Status**

4. Did money or worry about money affect your decision to come to clinic today? If so, how?

Probe: Does your family’s income or finances affect how you make decisions about seeking care when you are sick? If so, how?

Can you tell me about a decision you made in the past to not seek care for health problems because you were worried about money? What are your specific worries regarding money?

5. Does illness affect social status in your community? Do you think your situation in life is similar to most of the members of your community? Y/N

6. Do you see any differences in your community regarding how men and women use health care?

Probe

Would your decision to come to clinic today have been the same or different if you were a man/woman?

7. What kinds of support have you gotten from your community in the past when you have been sick? Can you tell me about it?

How have you helped someone else in your community who was sick or suffering? What groups in your community can people turn to for help when worried about having a really big change in their social or economic status?

Probe: What specific programs might help with this situation?

8. Have your symptoms or your visits to the clinic affected your ability to work? If so, how?

Probe: Are there ways that your employer or community makes it easier for you to work even with symptoms?

Have you been working less because of your symptoms or because of clinic visits?

**Material Circumstances**

9. What worries do you have for yourself or your family? Probe: Do you worry about having enough food for your family; education or other things for your children; transportation; your work or lack of work, your health?

10. Does seeking health care for your symptoms affect these worries?

**Psychosocial Circumstances**

11. How do most people in the community feel about others who get diseases like TB (may need to describe TB)? Probe: Is judgment by others a worry for people in your community when seeking care for cough or TB symptoms?

12. How do friends, family and community support you in your decision to seek care for your symptoms?

Probe: Do you have a strong social network? How do they support you? Do you ever feel socially isolated? y/n

How has your friend/family/community support affected your decisions about getting to clinic or accessing health care

for your symptoms?

13. How do people in your community react to your symptoms?

Probe: How do people in your community with similar symptoms manage work when they are getting tested for an illness like TB?

**Behavioral and Biological Factors**

14. What other health- related conditions do you have that you need treatment for?

Probes:

- Diabetes?
- Smoking?
- Drinking alcohol?
- Heart problems?
- Cancer?
- HIV?

15. How do these other medical conditions affect your ability to get care for your current symptoms

How do these other medical conditions affect your ability to be evaluated for an illness like TB?
